# Sphingosylphosphorylcholine inhibits plasma cell differentiation and ameliorates experimental autoimmune encephalomyelitis

**DOI:** 10.3389/fimmu.2023.1151511

**Published:** 2023-06-20

**Authors:** Byunghyun Park, Yu Sun Jeong, Wonseok Hu, Mingyu Lee, Ji Cheol Kim, Geon Ho Bae, Yong-Soo Bae, Yoe-Sik Bae

**Affiliations:** ^1^ Department of Biological Sciences, College of Science, Sungkyunkwan University, Suwon, Republic of Korea; ^2^ Department of Health Sciences and Technology, Samsung Advanced Institute for Health Sciences & Technology, Sungkyunkwan University, Seoul, Republic of Korea

**Keywords:** sphingosylphosphorylcholine, plasma cells, S1PR3, experimental autoimmune encephalomyelitis, multiple sclerosis

## Abstract

**Introduction:**

Multiple sclerosis (MS) is a potentially disabling disease that damages the brain and spinal cord, inducing paralysis of the body. While MS has been known as a T-cell mediated disease, recent attention has been drawn to the involvement of B cells in its pathogenesis. Autoantibodies from B cells are closely related with the damage lesion of central nervous system and worse prognosis. Therefore, regulating the activity of antibody secreting cell could be related with the severity of the MS symptoms.

**Methods:**

Total mouse B cells were stimulated with LPS to induce their differentiation into plasma cells. The differentiation of plasma cells was subsequently analyzed using flow cytometry and quantitative PCR analysis. To establish an experimental autoimmune encephalomyelitis (EAE) mouse model, mice were immunized with MOG^35–55^/CFA emulsion.

**Results:**

In this study, we found that plasma cell differentiation was accompanied by upregulation of autotaxin, which converts sphingosylphosphorylcholine (SPC) to sphingosine 1-phosphate in response to LPS. We observed that SPC strongly blocked plasma cell differentiation from B cells and antibody production *in vitro*. SPC downregulated LPS-stimulated IRF4 and Blimp 1, which are required for the generation of plasma cells. SPC-induced inhibitory effects on plasma cell differentiation were specifically blocked by VPC23019 (S1PR1/3 antagonist) or TY52159 (S1PR3 antagonist), but not by W146 (S1PR1 antagonist) and JTE013 (S1PR2 antagonist), suggesting a crucial role of S1PR3 but not S1PR1/2 in the process. Administration of SPC against an EAE mouse model significantly attenuated the symptoms of disease, showing decreased demyelinated areas of the spinal cord and decreased numbers of cells infiltrated into the spinal cord. SPC markedly decreased plasma cell generation in the EAE model, and SPC-induced therapeutic effects against EAE were not observed in μMT mice.

**Conclusion:**

Collectively, we demonstrate that SPC strongly inhibits plasma cell differentiation, which is mediated by S1PR3. SPC also elicits therapeutic outcomes against EAE, an experimental model of MS, suggesting SPC as a new material to control MS.

## 1 Introduction

Multiple sclerosis (MS) is a chronic autoimmune disease, characterized by demyelination of nerve cells in the spinal cord and brain, causing damage in communication between the brain and peripheral body (1). In the pathogenesis of MS, inflammatory cells such as T_H_1, T_H_17, memory B cells and plasma cells are recruited into the central nervous system (CNS), contributing to the development and promotion of inflammation in MS. Classically, MS has been regarded as a T cell-mediated disorder (2). However, accumulating evidence shows that B cells also play crucial roles in MS pathogenesis (3, 4). Moreover, recent successful clinical trials treating MS with anti-CD20 antibodies such as ocrelizumab and ofatumumab demonstrate that B cells could be effective targets to control MS (5, 6).

In MS, B cells show abnormal phenotypes and defective in regulatory functions. The abnormalities include the production of pro-inflammatory cytokines, antigen presentation to autoreactive T cells, the formation of follicle-like structure and defective regulatory functions. The immune cell infiltration, especially B cell migration into the meningeal regions is the pathological feature of the MS, which commonly correlates with a disease progression. Those infiltrated B cells form the tertiary lymphoid follicles containing various cell types such as T cells, dendritic cells, B cells, and plasma cells. The local follicle-like structures suggests that B cells in the follicles undergo activation and differentiation and support the disease progression (7). Not only antigen presentation to T cells and pro-inflammatory cytokine production, but also the production of auto-reactive antibodies from B cells is a key immune-dependent mechanism for damaging the CNS. In fact, 95% of MS patients have oligoclonal bands in the cerebrospinal fluid (CSF) derived from these auto-reactive antibodies (7). Additionally, the presence of IgM in the CSF has been linked to a more unfavorable prognosis in MS (8). Autoantibodies play a crucial role not only in the development of MS but also in other autoimmune disorders in CNS, such as myelin oligodendrocyte glycoprotein (MOG) antibody-associated disorder, anti-N-methyl-D-aspartate receptor encephalitis, and systemic lupus with neuropsychiatric manifestations (9). Consequently, the regulation of autoantibody production and plasma cell function holds the potential to alleviate symptoms associated with MS and various other autoimmune diseases.

Sphingosine 1-phosphate (S1P) receptor (S1PR) modulators represented by Fingolimod (FTY720) are one of the best targets for the treatment of MS and other autoimmune diseases (10). S1P is a bioactive signaling lipid known for its role in regulating immune cell egression from lymph nodes. S1PRs belong to the G protein coupled receptors and have five isoforms. The best studied S1PR is S1PR1 with the modulatory effect of lymphocyte migration from the lymphoid organs (10). S1P is produced by several enzymes and autotaxin is one of the major S1P producing enzymes in MS condition, which is exclusively detected in the CSF of MS patients, not other neurological diseases (11, 12). The expression and activity of the enzyme are elevated in MS condition (12). Autotaxin has lysophospholipase D activity, which converts lysophosphatidylcholine (LPC) and sphingosylphosphorylcholine (SPC) to lysophosphatidic acid (LPA) and S1P, respectively (13). Therefore, the elevated autotaxin may cause the imbalance in the ratio of SPC and S1P. The role of S1P is well known as leukocyte trafficking and S1PR modulators have impressive effect as therapeutics for MS, however, little is known regarding the functional roles of autotaxin and its substrate SPC in plasma cell differentiation in MS pathology.

This study aims to investigate the regulatory role of SPC in plasma cell differentiation, despite autotoxin is known to be elevated, and to assess its impact on MS pathology. We demonstrate that SPC strongly inhibits plasma cell differentiation, which is mediated by S1PR3. We also show that SPC effectively ameliorates against experimental autoimmune encephalomyelitis (EAE), suggesting SPC as a useful material to develop new drugs to control MS.

## 2 Materials and methods

### 2.1 Plasma cell differentiation

Splenocytes were isolated from C57BL/6 mice and negatively selected by the MagniSort™ mouse B cell enrichment kit (Thermo Fisher Scientific) to acquire total mouse B cells. Separated B cells were stimulated by LPS from *Escherichia coli* (10 μg/ml, Sigma-Aldrich) for 3 days. B cells were cultured in RPMI 1640 (Welgene) with 10% fetal bovine serum (FBS), 1% antibiotics (Thermo Fisher Scientific), and 50 μM β-mercaptoethanol (Sigma-Aldrich). CD138^+^B220^low^ plasma cells were analyzed by flow cytometry. IgM produced during plasma cell differentiation was measured by ELISA (Thermo Fisher Scientific). For *in vivo* plasma cell differentiation by LPS, LPS (0.6 mg/kg) was intravenously injected into 8-12 week-old C57BL/6 mice. On day 4 after LPS injection, mice were euthanized, and the plasma cell population in the splenocytes was analyzed using flow cytometry.

### 2.2 Flow cytometry

Cells were blocked by anti-mouse CD16/32 antibodies (Invivomab) before surface staining. Surface proteins expressed on cells were stained with fluorescence conjugated antibodies diluted in FACs buffer (1×PBS with 0.5% bovine serum albumin) at 4°C for 30 min. Live cells were stained by fixable viability dye (Thermo Fisher Scientific). Intracellular protein staining was performed by using the Intracellular Fixation & Permeabilization Buffer Set (Thermo Fisher Scientific), following the manufacturer’s recommended protocol. Intracellular proteins such as ENPP2 and IRF4 were stained after permeabilization. Anti-mouse IRF4 APC, anti-mouse Blimp-1 APC and anti-mouse B220 PE were purchased from Thermo Fisher Scientific. Anti-mouse CD138 APC was purchased from BD Biosciences. After staining, cells were washed and flow cytometry data acquired on FACScanto II (BD Biosciences), and the data was analyzed using FlowJo software (Tree Star).

### 2.3 Quantitative PCR

Cells were lysed by adding TRIzol reagent (Thermo Fisher Scientific) and chloroform was added to the lysed sample. RNA was isolated from the aqueous layer of chloroform and precipitated by using isopropyl alcohol. Extracted RNA was reversely transcribed into cDNA using Maxime RT premix (iNtRON Biotechnology). qPCR was performed on Qiagen real-time PCR instrument with 2X SYBR Green (BioFACT), and gene-specific primers: *Pax5*-forward, 5’-CCATCAGGACAGGACATGGAG-3’; *Pax5*-reverse, 5’-GGCAAGTTCCACTATCCTTTGG-3’; *Xbp1*-forward, 5’-AGCAAGTGGTGGATTTGGAAGA-3’; *Xbp1*-reverse, 5’-CACCAGCCTTACTCCACTCC-3’; *Prdm1*-forward, 5’-GCTGCTGGGCTGCCTTTGGA-3’; *Prdm1*-reverse, 5’-GGAGAGGAGGCCGTTCCCCA-3’; *Bcl6*-forward, 5’-ACCACCAGCCTCTTATCCCA-3’; and *Bcl6*-reverse, 5’-TGGGGACTCTGGGGACTAAC-3’, *Irf4*-forward, 5’-CTCTTCAAGGCTTGGGCATT-3’; *Irf4*-reverse, 5’-TGCTCCTTTTTTGGCTCCCT-3’, *Aicda*-forward, 5’-AAATGTCCGCTGGGCCAA-3’; *Aicda*-reverse, 5’-CATCGACTTCGTACAAGGG-3’, *Gapdh*-forward, 5’-TGCACCACCAACTGCTTAG-3’; *Gapdh*-reverse, 5’-GGATGCAGGGATGATGTTC-3’. *Tbp*-forward, 5’- AGAATAAGAGAGCCACGGACAA-3’; *Tbp*-reverse, 5’- CTTCACTCTTGGCTCCTGTG-3’. The qPCR data were quantified ΔCt values by using the 2-ΔΔCt method proportional to the expression of *Gapdh* and *Tbp* as internal controls.

### 2.4 EAE mouse model

8-12-week-old C57BL/6 female mice were purchased from Orient Bio Inc. B cell-deficient μMT mice were purchased from the Jackson Laboratory. The mice were housed 4-6 per cage in a clear and ventilated environment maintained under laboratory conditions (Temperature 22 ± 1°C, relative humidity 50 to 70%, and 12 h light and dark cycle). Standard food and water were provided throughout the experiments. Mice were acclimated to their surroundings over 5 days to eliminate the effect of stress prior to initiation of the experiments. Mice were immunized by MOG^35-55^/CFA emulsion, by injecting the emulsion subcutaneously (Hooke lab). At 2 h after immunization, pertussis toxin was intraperitoneally injected into mice at day 0 and 1 (100 ng/head). EAE mice were monitored for weight change and clinical scores were measured from day 7 after immunization. The weight of each mouse was measured by using an electronic scale and the clinical score was measured. After monitoring EAE, mice were sacrificed for analysis at day 19. Plasma cell populations in the spleen and lymph nodes were analyzed by flow cytometry.

### 2.5 Immunohistochemistry and tissue staining

Spinal cords were fixed and processed in paraffin blocks for histological analysis. Tissues were sectioned horizontally at 5 μm. Sectioned samples on the slides were deparaffinized and hydrated by incubating slides in xylene for 15 min, 3 times, and 100% and 95% ethanol for 5 min, 2 times each. Then, an antigen unmasking procedure was conducted by boiling in 10 mM sodium citrated buffer (pH 6) for 10 min. Mouse-/rabbit-specific HRP/DAB (ABC) detection IHC kit (Abcam) was used following the manufacturer’s recommended protocol to develop slides by the DAB substrate. Anti-mouse CD138 and anti-rabbit CD4 polyclonal antibodies for use as primary antibodies were purchased from Thermo Fisher Scientific. Infiltrated cells and myelinated area were measured by hematoxylin & eosin (H&E) staining and luxol fast blue staining, followed by using ImageJ. Luxol fast blue staining was conducted by using the Luxol Fast Blue Stain Kit (Abcam), following the manufacturer’s recommended protocol. Representative histological images were selected for each group of mice.

### 2.6 Carboxyfluorescein diacetate succinimidyl ester tagged proliferation assay

Cells were stained by 2 μM of CFSE (Thermo Fisher Scientific) in PBS supplemented with 3% FBS. The cells were incubated for 20 min at room temperature. Cells were washed by PBS with 5% FBS twice. Then, the cells were incubated for 1 h with RPMI 1640 supplemented with 10% FBS and 1% antibiotics for stabilization of CFSE. After administration of proper stimulation, the intensity of CFSE was measured by flow cytometry.

### 2.7 Statistical analysis

GraphPad Prism software was used to evaluate results. All results are expressed as the mean ± SEM for the data obtained from the indicated number of experiments. Statistical analysis was performed using Student’s *t*-test or one-way-ANOVA, or two way-ANOVA. A *P* value ≤ 0.05 was considered statistically significant.

## 3 Results

### 3.1 SPC suppresses plasma cell differentiation

**Figure 1:**
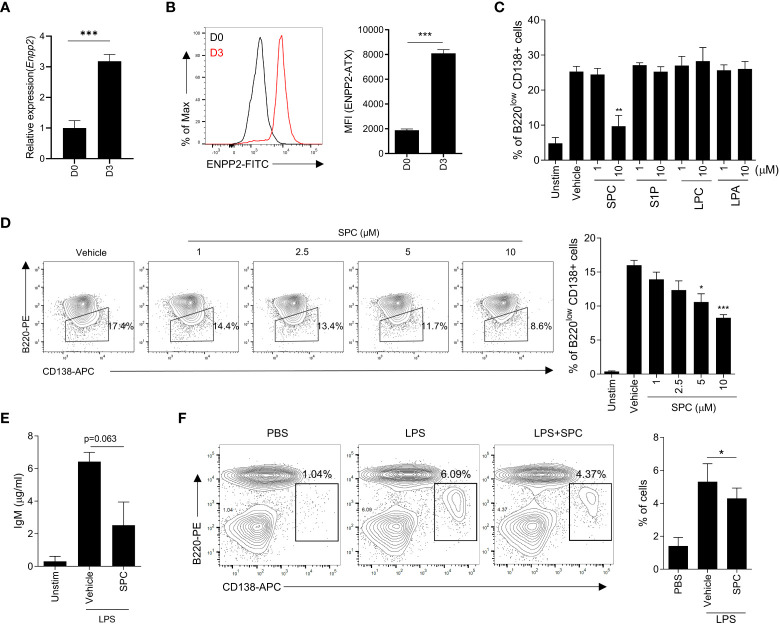
SPC inhibits plasma cell differentiation *in vitro*. **(A–D)** Total B cells from the splenocytes of C57BL/6 mice were stimulated by LPS (10 μg/ml) for 72 h. **(A, B)** ENPP2 (autotaxin) expression at day 0 (D0) and day 3 (D3) was measured by qPCR **(A)** and flow cytometry **(B)**. **(C)** Plasma cell populations (B220^low^CD138^+^) in response to LPS in the presence of vehicle, 10 μM of SPC, S1P, LPC, or LPA were measured by flow cytometry. **(D)** Levels of plasma cells in response to LPS in the presence of several concentrations of SPC (1, 2.5, 5, 10 μM) were measured by flow cytometry. **(E)** IgM levels were measured by ELISA. **(F)** LPS (0.6 mg/kg) was intravenously injected at 8-12 week-old C57BL/6 mice for *in vivo* plasma cell differentiation. Mice were sacrificed at day 4 after injecting LPS. SPC (4 mg/kg) was injected daily during experiments. Plasma cell population (B220^low^ CD138^+^) was analyzed by flow cytometry. Representative histogram or dot plot data are shown (B left, D left and F left). Data are expressed as the mean ± SEM (n=3-4 for A, B right, C, D right, E, F right). *P* values were calculated by Student’s *t*-test **(A–C, E, F)** and one-way ANOVA with Tukey’s multiple comparisons test **(D)**.*P < 0.05, **P<0.01, ***P < 0.001.

This study aimed to elucidate the impact of SPC on MS pathology, with a particular focus on plasma cells as a crucial cell type in disease progression. Furthermore, we aimed to investigate the impact of SPC under conditions of elevated autotaxin levels, which mimics the inflammatory condition and MS. The expression of autotaxin is increased with proinflammatory stimulation including tumor necrosis factor, IL-6, and LPS (13). Moreover, previous reports demonstrated that the expression of autotaxin is augmented in several cell types including B cells in autoimmune disorders (13, 14). Therefore, we differentiated B cells with LPS stimulation and examined the level of autotaxin. Stimulation of isolated B cells with LPS for 3 days induced plasma cell differentiation, and the gene expression of *Enpp2* encoding autotaxin was strongly increased during the differentiation induced by LPS (**Figure 1A**). Flow cytometric analysis also revealed that LPS significantly increased the expression of autotaxin (**Figure 1B**).

Autotaxin converts SPC or LPC into S1P or LPA with its lysophospholipase D activity (15). Our finding on the upregulation of autotaxin during plasma cell differentiation led us to examine whether plasma cell differentiation is affected by the substrates of autotaxin (SPC or LPC) or the products of autotaxin (S1P or LPA). We found that SPC can downregulate plasma cell differentiation but the other three lipids (S1P, LPC and LPA) did not affect LPS-induced plasma cell differentiation (**Figure 1C**). Stimulation of B cells with LPS for 3 days significantly increased the level of plasma cells which are B220^low^CD138^+^ (**Figure 1C**). Addition of 10 μM SPC significantly decreased the level of B220^low^CD138^+^ cells in the presence of LPS (**Figure 1C**), suggesting that SPC inhibits plasma cell differentiation induced by LPS. In separate experiments, we found that all the tested bioactive lipids had no effect on the viability of B cells (**Supplementary Figure 1**). Therefore, we can rule out the possibility that SPC inhibits plasma cell differentiation by increasing cell death. We found that SPC inhibited plasma cell differentiation induced by LPS in a concentration-dependent manner, showing significant inhibitory effects at 5-10 μM (**Figure 1D**). Plasma cell differentiation is associated with increased secretion of IgM (16). We also found that LPS stimulation of B cells significantly increased secreted IgM level, which was markedly decreased by SPC (**Figure 1E**).

Next, we examined whether SPC affects plasma cell differentiation *in vivo*. Administration of LPS (0.6 mg/kg) for 4 days increased B220^low^CD138^+^ cells in the spleen, suggesting that LPS injection increased plasma cell differentiation *in vivo* (**Figure 1F**). Daily subcutaneous injection of SPC for 4 days significantly decreased the level of LPS-induced plasma cell differentiation in the spleen in the LPS-injection model (**Figure 1F**). This result supports our hypothesis that SPC is a new molecule that inhibits plasma cell differentiation.

### 3.2 SPC downregulates IRF4 expression

**Figure 2:**
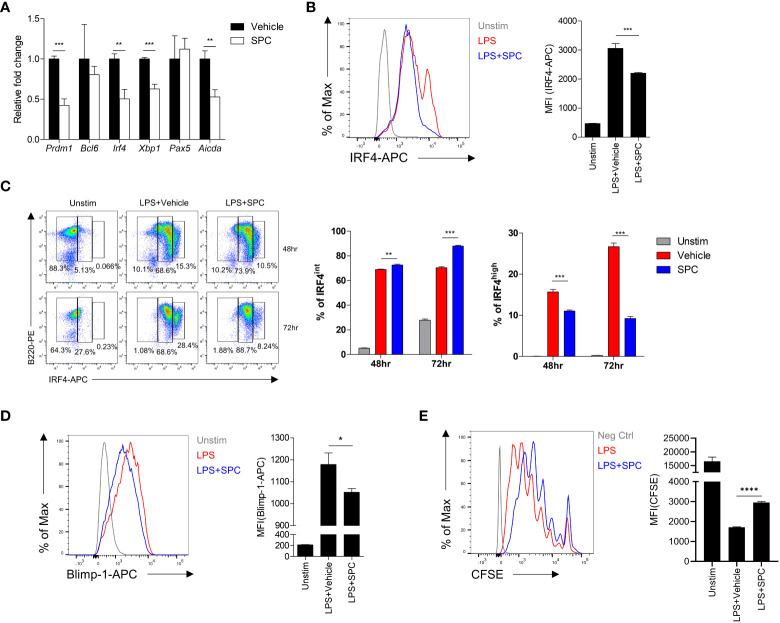
SPC downregulates IRF4 and Blimp-1 to suppress plasma cell differentiation. **(A-D)** Total B cells from the splenocytes of C57BL/6 mice were stimulated by 10 μg/ml LPS. **(A)** Indicated gene mRNA expression was measured by qPCR at 72 h after differentiation. **(B)** IRF4 expression at 72 h after LPS stimulation was measured by flow cytometry. Representative histogram is shown (left). **(C)** IRF4^int^ and IRF4^high^ population at the indicated time points were compared between LPS + vehicle- and LPS + SPC (10 μM)-treated group. **(D)** Levels of Blimp-1 were compared between LPS + vehicle- and LPS + SPC (10 μM)-treated group. **(E)** Cell proliferation was analyzed with CFSE labeling by flow cytometry. Levels of CFSE were compared between LPS + vehicle- and LPS + SPC (10 μM)-treated group. Data are expressed as the mean ± SEM (n=3-4 for A, B right, C right, D right, E right). *P* values were calculated by Student’s *t*-test. *P<0.05, **P<0.01, ***P<0.001, ****P<0.0001.

Plasma cell differentiation is associated with changes of gene expression pattern in B cells (17–20). Addition of SPC during plasma cell differentiation induced by LPS significantly decreased the expression levels of several plasma cell-associated genes such as *Prdm1*, *Irf4*, *Xbp1* (**Figure 2A**). Xbp-1 is a downstream signaling molecule of Blimp-1 encoded by *Prdm1*. Besides, *Aicda* is related to class switch recombination of B cells. Among the plasma cell-associated genes, *Irf4* and *Prdm1* are known as key transcription factors that mediate plasma cell differentiation (20). We found that stimulation of B cells with LPS for 3 days strongly upregulated the level of IRF4 in the cells, which was significantly decreased by SPC (**Figure 2B**). This result is consistent with our finding that SPC inhibits plasma cell differentiation. We further examined the effects of SPC on the expression of IRF4 during plasma cell differentiation. Stimulation of B cells with LPS generated two IRF4-positive populations (IRF4^int^ and IRF4^high^) by decreasing the IRF4^negative^ population in a time-dependent manner. Addition of SPC significantly increased the IRF4^int^ population, while strongly decreasing the IRF4^high^ population (**Figure 2C**). A previous report demonstrated that the IRF4^high^ population differentiates into plasma cells in response to outside stimuli (20). Moreover, we found that Blimp-1 was also decreased by SPC, which is another key transcription factor of plasma cell differentiation (**Figure 2D**). In conclusion, SPC downregulates plasma cell differentiation, which is accompanied by decrease of IRF4 and Blimp-1 levels in B cells after activation. During the differentiation to plasma cells, B cells undergo multiple divisions to receive the necessary signals for regulating key transcription factors, such as IRF4 and Blimp-1 (21). We observed that the addition of SPC during LPS-induced plasma cell differentiation significantly reduced cell proliferation compared to the vehicle (**Figure 2E**). The inhibitory effect of SPC on B cell proliferation suggests its impact not only on suppressing plasma cell differentiation but also on modulating B cell activation.

### 3.3 SPC downregulates plasma cell differentiation through S1PR3 signaling

**Figure 3:**
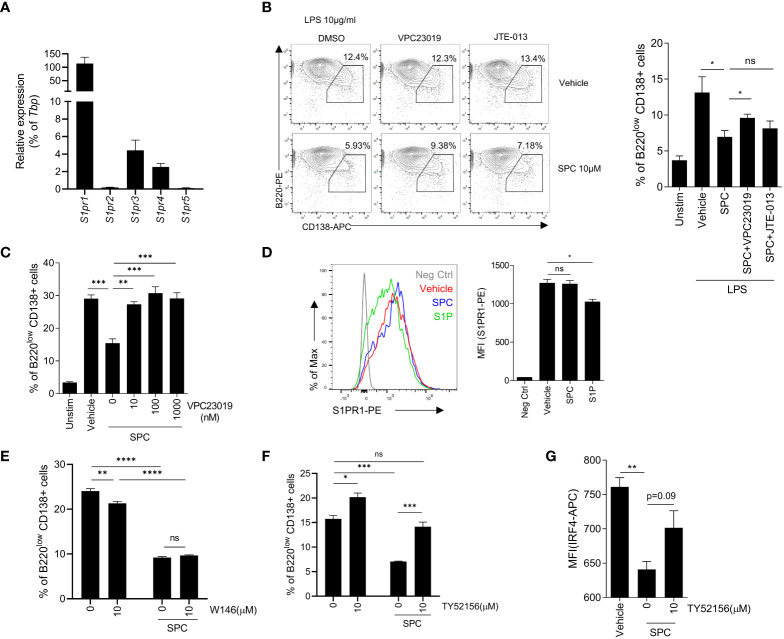
SPC inhibits plasma cell differentiation via S1PR3 signaling. **(A–E)** Total B cells from the splenocytes of C57BL/6 mice were stimulated by LPS (10 μg/ml). **(A)** mRNA expression of indicated S1P receptors were measured by qPCR. **(B)** Plasma cell populations (B220^low^ CD138^+^) at 72 h were compared in unstimulated, LPS + vehicle, LPS + SPC, LPS + SPC + VPC23019 (100 nM) and LPS + SPC + JTE-013 (100 nM) cells. Representative dot plots of VPC23019 and JTE-013 treated groups are shown (left). **(C)** Plasma cell populations at 72 h were compared between unstimulated, LPS + vehicle, LPS + SPC (10 μM) + VPC23019 (0, 10, 100, 1000 nM) cells. **(D)** S1PR1 expression was compared at 72 h after stimulation in negative control, LPS + vehicle, LPS + SPC (10 μM), LPS + S1P (10 μM) cells. **(E)** Plasma cell population at 72 h were compared in LPS + vehicle, LPS + SPC (10 μM) + W146 (0, 10 μM) cells. **(F)** IRF4 expression in LPS + vehicle, LPS + SPC (10 μM) + TY52156 (0, 10 μM) cells were measured. Representative histogram or dot plot data were shown (B left and D left). Data are expressed as the mean ± SEM (n=3 for A, B right, C, D right, E, F, G). *P* values were calculated by Student’s *t*-test **(B, D, G)** and one-way ANOVA with Tukey’s multiple comparisons test **(C)**, and two-way ANOVA with Sidak’s multiple comparisons test **(E, F)**. *P < 0.05, **P<0.01, ***P < 0.001, ****P < 0.0001. ns, not significant.

Because of the similarity of the structure of sphingolipid, SPC acts on several S1PRs such as S1PR1, S1PR2, S1PR3, and so on (22–24). Thus, we focused on S1PRs to identify the target receptor(s) of SPC that mediates the inhibitory effects of SPC on plasma cell differentiation induced by LPS. First, we examined whether B cells express S1PRs during activation. We found that S1PR1, S1PR3, and S1PR4 expression are relatively high compared to S1PR2 and S1PR5 at the naïve state of B cells (**Figure 3A**). We next investigated whether SPC acts on S1PRs to block plasma cell differentiation using two different S1PR antagonists: VPC23019 as an S1PR1/3 antagonist, JTE-013 as an S1PR2 antagonist. We found that the SPC-induced inhibitory effects on plasma cell differentiation were significantly attenuated by VPC23019 but not by JTE-013 (**Figure 3B**). Addition of several different concentrations of VPC23019 significantly alleviated SPC-induced inhibition of plasma cell differentiation in response to LPS in a concentration-dependent manner (**Figure 3C**). Fingolimod (FTY720), an FDA-approved immunosuppressive drug for the treatment of MS, mainly acts on S1PR1 (25). It is known that S1PR1 specific modulators (S1P or FTY720) internalize S1PR1 with receptor binding. We found that stimulation of B cells with S1P elicited S1PR1 internalization with flow cytometric analysis (**Figure 3D**). However, SPC did not induce S1PR1 internalization (**Figure 3D**), suggesting that SPC may not act through S1PR1. Moreover, the S1PR1 specific antagonist, W146, did not block the suppressive effect of SPC on the plasma cell differentiation induced by LPS (**Figure 3E**). On the other hand, TY52156, another S1PR3 antagonist, significantly blocked the inhibitory actions of SPC on plasma cell differentiation (**Figure 3F**). Moreover, TY52156 reversed the downregulating effects of SPC on IRF4 (**Figure 3G**). Collectively, our results suggest that SPC may act on S1PR3 but not S1PR1 or S1PR2 to block plasma cell differentiation in response to LPS.

### 3.4 SPC alleviates EAE with the decreased accumulation of plasma cell in the CNS

**Figure 4:**
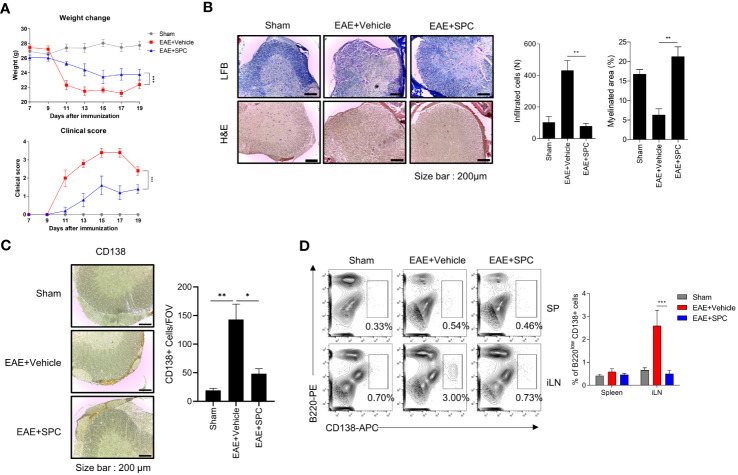
SPC attenuates EAE by suppressing plasma cell formation. **(A–C)** 8-12 week-old female C57BL/6 mice were immunized with MOG^35-55^/CFA emulsion. SPC (4 mg/kg) was administrated subcutaneously daily. **(A)** Weight change and clinical score were monitored for 19 days. **(B–D)** Mice were sacrificed at 19 days after immunization. **(B)** Spinal cord sections were stained with luxol fast blue (LFB) and H&E (×100). Representative images are shown. **(C)** Spinal cord sections were stained with anti-CD138 antibody for immunohistochemistry. Representative images are shown. **(D)** Plasma cell populations in the spleen and inguinal lymph nodes were compared in sham, EAE + vehicle, EAE + SPC (4 mg/kg) mice. Representative dot plot data are shown. Data are expressed as the mean ± SEM (n=5 for A, B right, D right, n-=3 for C right). *P* values were calculated by Student’s *t*-test **(B, C)** or two-way ANOVA with Tukey’s multiple comparisons test **(A)**, or two-way ANOVA with Sidak’s multiple comparisons test **(D)**. *P<0.05, **P<0.01, ***P < 0.001.

Plasma cells have been regarded to be important players in the development and progression of autoimmune disorders including MS (26, 27). Since we found that SPC strongly blocks plasma cell differentiation, we investigated whether SPC shows beneficial effects against an EAE model, an animal model of MS. Establishment of the EAE model caused the decrease of gradual body weight and the increase of clinical score. However, daily subcutaneous injection of SPC significantly decreased the clinical score and led to a recovery of body weight (**Figure 4A**). Luxol fast blue staining showed that EAE establishment markedly increased demyelination in the spinal cord, which was almost completely blocked by SPC administration (**Figure 4B** top). Immune cell infiltration, analyzed by H&E staining, was induced by EAE establishment. SPC strongly blocked immune cell infiltration into the spinal cord (**Figure 4B** bottom). Previous reports demonstrated that EAE pathogenesis is associated with CD138^+^ plasma cells in the CNS (26, 28). We also found that CD138^+^ cells were highly increased in the meningeal area of the spinal cord in EAE model mice. SPC administration markedly blocked the accumulation of CD138^+^ cells (**Figure 4C**). In addition to reducing CD138^+^ cell population, the administration of SPC also led to a decrease in the infiltration of CD4 lymphocytes into the spinal cord (**Supplementary Figure 2**). These results suggest that SPC effectively inhibits plasma cell infiltration into the CNS.

Next, we examined the effects of SPC on plasma cell differentiation in the EAE model. Establishment of the EAE model significantly increased B220^low^CD138^+^ cell levels in the inguinal lymph node but not in the spleen. Administration of SPC significantly decreased the levels of the B220^low^CD138^+^ cell population to the basal level (**Figure 4D**). In addition, when we induced the EAE model in μMT B cell-deficient mice and injected SPC, SPC did not show therapeutic effects (**Supplementary Figure 3**). These results suggest that B cells are important for the SPC-induced therapeutic effects on the EAE model. In conclusion, the results suggest that SPC may show beneficial effects against the EAE model with decreased plasma cell differentiation in peripheral lymph nodes and the infiltration into the CNS.

### 3.5 SPC-induced therapeutic effects against EAE is blocked by TY52156

**Figure 5:**
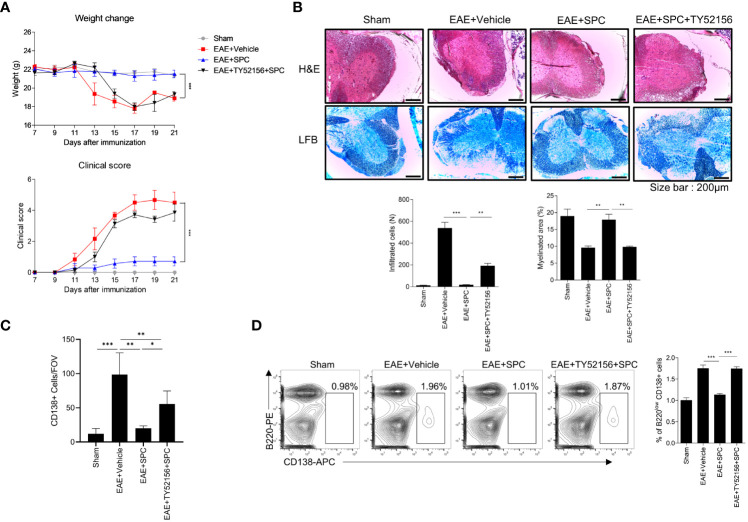
SPC-induced therapeutic effects against EAE are blocked by TY52156. 8-12 week-old female C57BL/6 mice were immunized with MOG^35-55^/CFA emulsion. TY52156 (5 mg/kg) was subcutaneously injected in 2-day intervals and SPC (4 mg/kg) was subcutaneously injected daily. **(A)** Weight change and clinical score were monitored for 21 days. **(B-C)** Mice were sacrificed at 21 days after immunization. **(B)** Spinal cord sections were stained with luxol fast blue (LFB) and H&E (×100). Representative images are shown. **(C)** Spinal cord sections were stained with anti-CD138 antibodies for immunohistochemistry. **(D)** Plasma cell population in inguinal lymph nodes were compared in sham, EAE + vehicle, EAE + SPC (4 mg/kg), EAE + TY52156 (5 mg/kg) + SPC (4mg/kg) mice. Data are expressed as the mean ± SEM (n=6-7 for A, n=4-7 for B, D, n=3 for C). *P* values were calculated by Student’s *t*-test **(B–D)** or two-way ANOVA with Tukey’s multiple comparisons test **(A)**. *P < 0.05, **P<0.01, ***P < 0.001.

Since plasma cells have an important role in the production of autoantibodies (29, 30) and SPC downregulates plasma cell differentiation via S1PR3 (**Figure 3C**), we subsequently investigated whether an S1PR3 antagonist affects the therapeutic effects of SPC on the EAE model. Administration of TY52156 (an S1PR3 antagonist) significantly blocked the therapeutic effects of SPC in the EAE model (**Figure 5A**). TY52156 administration blocked the beneficial effects of SPC on the EAE model in terms of infiltration of immune cells in the spinal cord, demyelination, and plasma cell population (**Figures 5B, C**) and peripheral plasma cell population (**Figure 5D**). In separate experiments, we compared the effects of SPC and FTY720 (an FDA-approved MS therapeutic drug, Fingolimod) on the generation of plasma cells in the EAE model. Unlike SPC, FTY720 failed to decrease plasma cell differentiation *in vitro* (**Supplementary Figure 4A**). Administration of FTY720 also did not attenuate plasma cell differentiation in draining lymph nodes of EAE model mice (**Supplementary Figure 4B**). Collectively, SPC seems to exhibit therapeutic effects to treat EAE through mechanisms different from FTY720 in terms of target receptor and effect on plasma cell differentiation.

## 4 Discussion

Autotaxin is a secreted glycoprotein that exhibits lysophospholipase D activity, converting SPC and LPC to S1P and LPA, respectively. Its expression is induced by proinflammatory factors such as LPS, tumor necrosis factor, and IL-6, as well as chronic inflammatory conditions like hepatitis, arthritis, and MS (13). Notably, autotaxin is exclusively detected in the CSF of MS patients but not of other neurological diseases patients, and its activity is elevated in individuals with MS (11, 12). In fact, the role of autotaxin in MS is still controversial. Therapeutic administration of an LPA (the product of autotaxin) receptor agonist reduced clinical signs of EAE (31).On the contrary, genetic deletion of autotaxin from CD11b^+^ cells was reported to decrease EAE severity (32). Despite of the controversial effect of autotaxin and its product signaling, it is clear fact that the level of autotaxin is elevated in MS patients and the inhibition of S1P, another product of the enzyme, shows significant therapeutic effect on MS. The goal of this investigation was the determination of the effect of SPC, the substrate of autotaxin, on the differentiation of plasma cell and EAE animal model. In this study, we found that stimulation of B cells with LPS strongly increased the expression of autotaxin (**Figures 1A, B**). We examined the effects of two autotaxin substrates (SPC and LPC) and two autotaxin products (S1P and LPA) on plasma cell differentiation. We found that only SPC, but not LPC, S1P, or LPA, significantly suppressed plasma cell differentiation from B cells (**Figure 1**). Subsequently, we also demonstrated that SPC administration strongly elicits therapeutic effects against EAE, an experimental MS model (**Figure 4**). Based on our results, we suggest that the bioactive lipid SPC elicits anti-EAE effects.

In the pathology of MS, the involvement of B cells is closely associated with disease progression. The success of anti-CD20 antibody therapy and the accelerated disease progression of MS patients with B cell-biased immune cell population in the CNS supports the importance of B cells in MS (7). B cells have several roles in the CNS inflammation including antibody production, antigen presentation to CD4 T cells, pro-inflammatory cytokine production, and immune response regulation. Among them, antibody production is the direct way of B cell involvement to damage to the CNS (9). The antibodies are detected in the active lesion and the CSF of MS patients. However, B cells are not detected within the directly damaged regions in MS. Instead, they accumulate at the entry points of CNS, specifically in the meningeal and barrier regions (8, 9). Notably, these accumulated B cells give rise to ectopic follicle-like structures that resemble lymphoid organs, which have been identified in the meninges. While the exact function of these structures remains controversial and unknown, studies have reported a correlation between their presence and disease severity. Furthermore, accumulating evidence indicates that B cells within these structures become activated, undergo differentiation, and interact with T cells (8). Like other lymphoid structures, these follicle-like B cell aggregates also contain various types of immune cells including CD8 T cells, CD4 T cells, dendritic cells, macrophages, B cells and plasma cells. Among these cell types, plasma cells are notably abundant within the follicles (9, 27, 33). Furthermore, the location of these structures in proximity to blood vessels facilitates the recruitment of immune cells into the CNS and the active site of inflammation (8). Considering the correlation between plasma cells and the follicle-like structure, we hypothesize that the inhibitory effect of SPC on the differentiation of plasma cells would also affect the B cell aggregates. We observed the significant decrease of CD138^+^ cell accumulation in the meninges of SPC injected mice, which supports our hypothesis. Our data suggest that SPC ameliorates the symptoms of EAE by not only suppressing plasma cell differentiation but also inhibiting the B cell accumulation in the CNS region. Previous studies demonstrated that B cells mediate pathogenic progress of MS via antigen presenting activity (34, 35). While this study did not investigate the impact of SPC on the antigen presenting cell function of B cells, it is crucial to explore whether SPC can indeed exert a therapeutic effect on EAE through the regulation of B cell antigen presenting cell function in the future.

To reveal the target receptor of SPC for the inhibitory effects on plasma cell differentiation, we used several antagonists for S1PRs. Because JTE013 failed to block the inhibitory effects of SPC on plasma cell differentiation, we could rule out the possible engagement of S1PR2. However, VPC23019 (a dual antagonist for S1PR1 and S1PR3) and TY52156 (an S1PR3 antagonist) effectively blocked the inhibitory effects of SPC on plasma cell differentiation. (**Figures 3B, C, F**). With this pharmacological approach, we assumed that S1PR1 or S1PR3 is involved in the inhibitory effects of SPC on plasma cell differentiation. To further test the functional role of S1PR1 on the SPC-induced response, we examined whether SPC affects S1PR1 activation in B cells by monitoring S1PR1 internalization with flow cytometry. Similar to sphingosine, FTY720 is initially phosphorylated by sphingosine kinase in the cells. Phospho-FTY720 works through S1PRs by internalizing and disabling these receptors by degradation (36). The disabling of the S1PRs ensures that immune cells do not migrate to blood or lymph which have a high S1P concentration. The activation of S1PR1 by its specific agonist (S1P) is mediated by the internalization of S1PR1, but SPC treatment did not result in internalization of S1PR1 (**Figure 3D**). Moreover, W146 (an S1PR1 antagonist) did not affect SPC-inhibited plasma cell differentiation (**Figure 3E**). Therefore, we could exclude the possible action of S1PR1 in the inhibitory activity on plasma cell differentiation. We conducted experiment to determine if S1PR3 antagonist could reverse the therapeutic effects of SPC against EAE, as observed in plasma cell differentiation. As shown in **Figure 5**, TY52156 significantly blocked the beneficial outcomes induced by SPC in the EAE model. In separate experiment, we found that the administration of TY52156 alone demonstrated amelioration of EAE symptoms (**Supplementary Figure 5**). Owing to the intricate effects of TY52156, the mechanism by which SPC treats EAE, specifically involving S1PR3, remains uncertain and needs further investigation in future studies. We also found that SPC and FTY720 have different protective mechanisms of action in the EAE model in terms of plasma cell generation *in vivo* (**Supplementary Figure 4**). SPC effectively blocked plasma cell differentiation whereas FTY720 did not. Our findings show a novel insight into SPC as a useful molecule to develop therapeutic drugs against MS with different mode of action of FTY720.

S1PR modulators represented by Fingolimod (FTY720), were approved for MS. S1PRs are G protein coupled receptors expressed on cell surfaces and throughout the body. S1P and S1PR signaling is well studied in cardiovascular system and lymphocyte trafficking (10, 37). S1PR modulation via functional antagonists or agonists regulate immune responses via inhibition of the egress of lymphocytes from the lymphoid organs. S1PR1 is the most studied and favored target for regulating lymphocytes. However, compared to S1PR1, other S1PR subtypes are less studied. SPC has known to acts on several S1PRs such as S1PR1, S1PR2, S1PR3, and so on because of the similarity of the structure of sphingolipid (22–24). In this study, we demonstrated that the inhibitory effect of plasma cell differentiation induced by SPC is mediated by S1PR3. S1PR3 signaling is complicated because of its diverse options for G_α_ subunit. S1PR3 can couple with G_αi/o_, G_αq_ and G_α12/13_ and convey signals with context dependent manner, whereas S1PR1 only couples with G_αi/o_ (38, 39). Most of the studies with the role of S1PR3 did not identify the G_α_ subunit in their experimental conditions. Unfortunately, we also did not investigate the specific G_α_ subunit through which SPC exerts its effects, adding to the complexity of S1PR3 signaling. This limitation highlights the need for further research to elucidate the precise signaling mechanisms involved. The context-dependent mechanism of S1PR3 also observed in our study. The administration of the S1PR3 antagonist alone demonstrated amelioration of EAE symptoms (**Supplementary Figure 5**), which contrasts with our findings using SPC and TY52156 (**Figure 5**). According to previous studies have shown abundant expression of S1PR3 in the nervous system, particularly in the dorsal root ganglion and astrocytes (38). Inflammatory signals increase S1PR3 levels in astrocytes, and astrocytic gliosis is observed in EAE and MS (38, 40). S1PR3 antagonists have been shown to provide protective effects in various neurological injuries, including cerebral infarction and spinal cord injury (39, 41). Therefore, we assume that the direct protective effect of TY52156 on the nervous system whereas the inhibitory effect of TY52156 on the effect of SPC with antagonistic role in the B cells.

In conclusion, we demonstrate that the effect of SPC on the plasma cell differentiation and EAE animal model. Mechanistically, SPC decreased the plasma cell differentiation induced by LPS. In an EAE model, SPC elicited significant therapeutic effects against the autoimmune disease. We also revealed that the molecular target of SPC is S1PR3, with inhibitory effects on plasma cell differentiation. This study elucidated the effect of SPC on plasma cell differentiation and MS pathogenesis, contributing to a better understanding of influence of SPC on the immune system.

## Data availability statement

The original contributions presented in the study are included in the article/**Supplementary Material**. Further inquiries can be directed to the corresponding author.

## Ethics statement

The animal study was reviewed and approved by Institutional Review Committee for Animal Care and Use at Sungkyunkwan University.

## Author contributions

BP, YJ, ML and Yoe-SB: conception and design of study. BP, YJ, WH, ML, YJ, JK, and GB: acquisition of data. BP, YJ, WH, and ML: analysis and interpretation of data. BP, YJ, Yong-SB and Yoe-SB: writing and review of the paper. All authors contributed to the article and approved the submitted version.
