# Supplementary material for: Sphingosylphosphorylcholine inhibits plasma cell differentiation and ameliorates experimental autoimmune encephalomyelitis
Source: Front Immunol. 2023 Jun 20;14:1151511. doi: 10.3389/fimmu.2023.1151511 (PMC10319473; doi:10.3389/fimmu.2023.1151511)
Supplement: Supplementary file 1 [file DataSheet_1.docx]

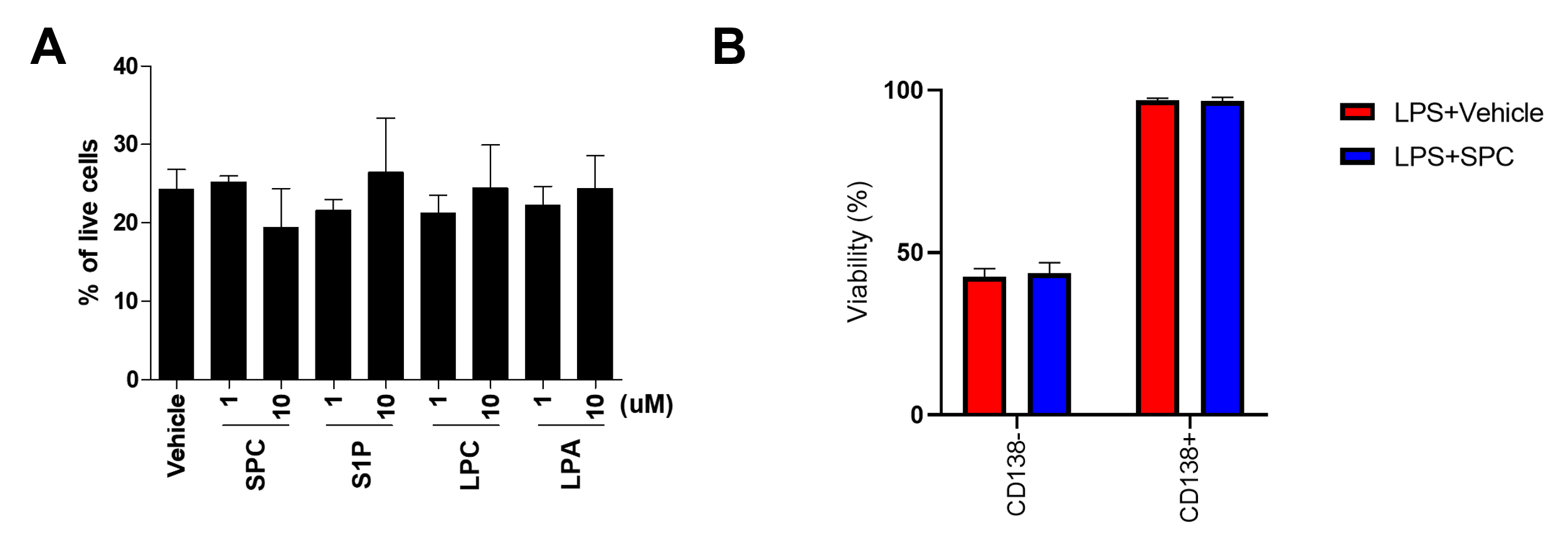
**Supplementary Figures**

**Supplementary Fig. 1. Viability and proliferation of B cells after LPS stimulation.** (A) Mouse B cells were stimulated by 10 μg/ml of LPS with vehicle, 10 μM of SPC, S1P, LPC and LPA. % of live cells were measured by staining with fixable viability dye. (B) Mouse B cells were stimulated by 10 μg/ml of LPS with vehicle and 10 μM of SPC. Viability of B cells were measured by staining with fixable viability dye and CD138 staining.


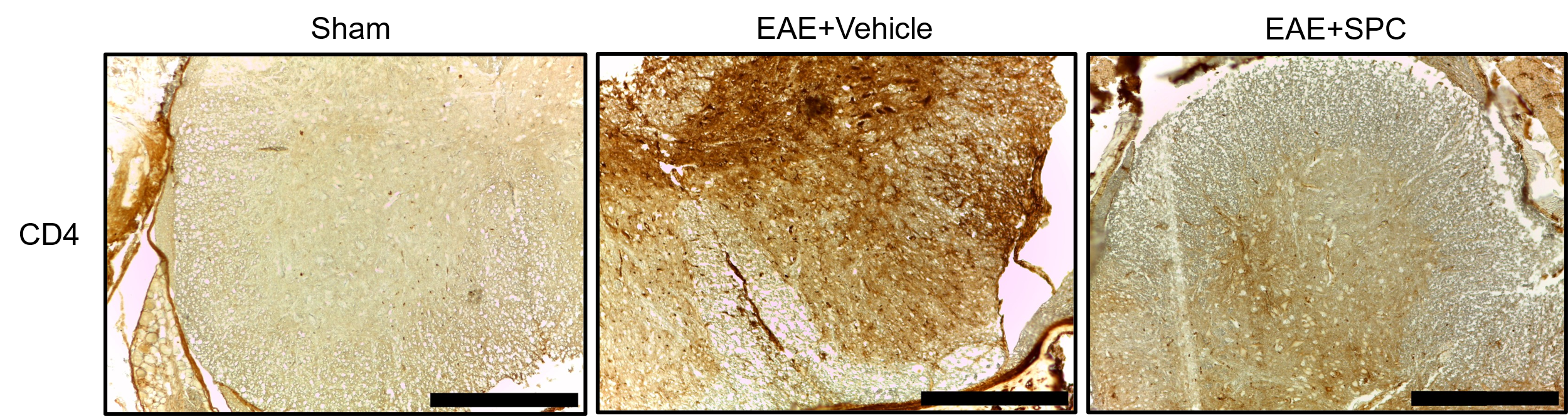


**Supplementary Fig. 2. SPC inhibits CD4+ cell infiltration into the spinal cord.** 8-12 week-old female C57BL/6 mice were immunized with MOG^35-55^ / CFA emulsion. SPC (4 mg/kg) was administrated subcutaneously daily. Mice were sacrificed at 19 days after immunization. Spinal cord sections were stained with anti-CD4 antibody for immunohistochemistry. Representative images are shown.


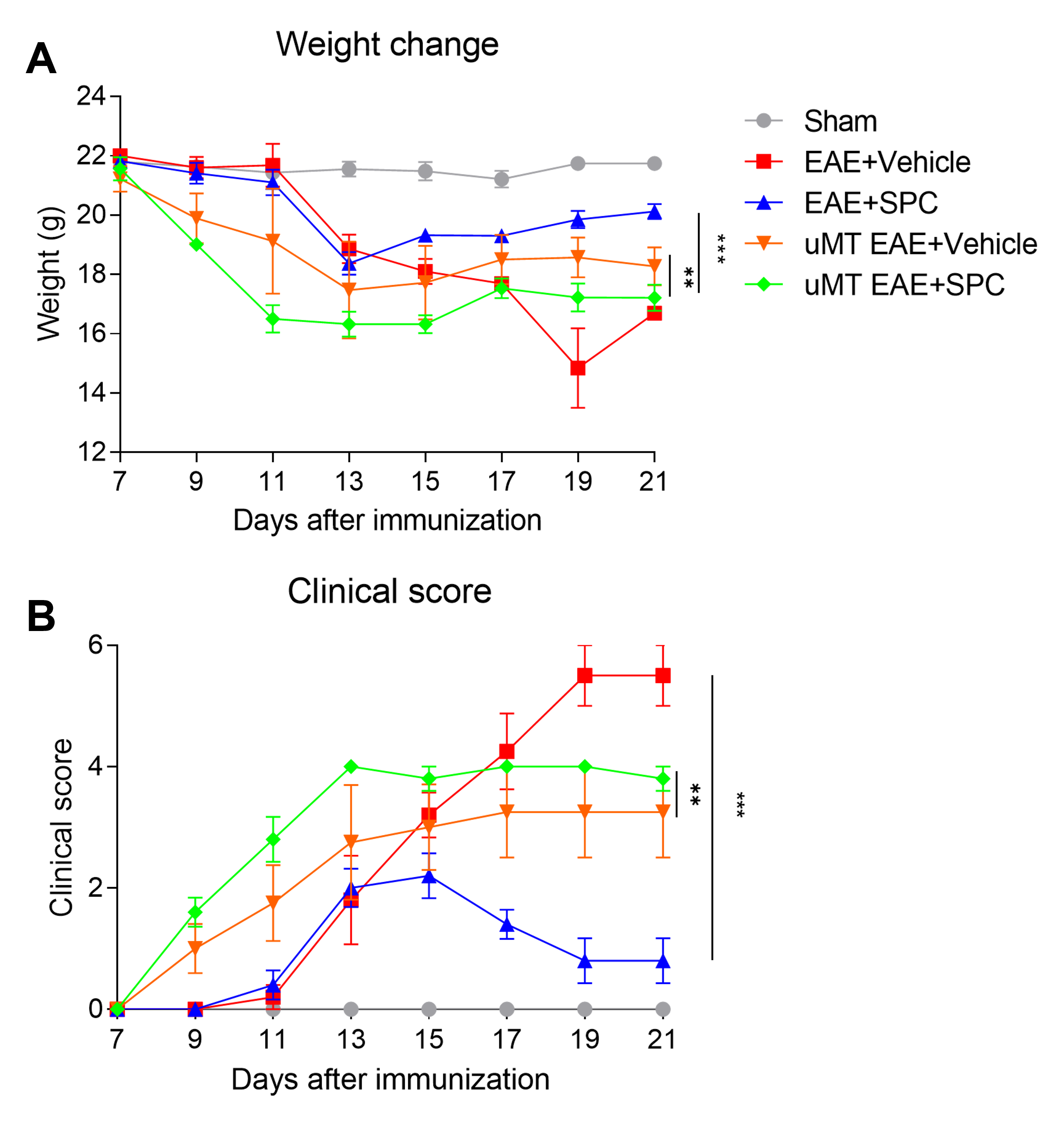


**Supplementary Fig. 3. SPC does not show therapeutic effects on EAE in μMT mice.** 8-12 week-old female C57BL/6 mice and μMT were immunized with MOG^35-55^ / CFA emulsion. Vehicle (PBS) and SPC (4 mg/kg) was subcutaneously injected daily. Weight change and clinical score were monitored for 21 days. Data are expressed as the mean ± SEM (n=4-5). *P* values were calculated by two-way ANOVA with Tukey’s multiple comparisons test. ***P* < 0.01, ****P* < 0.001.


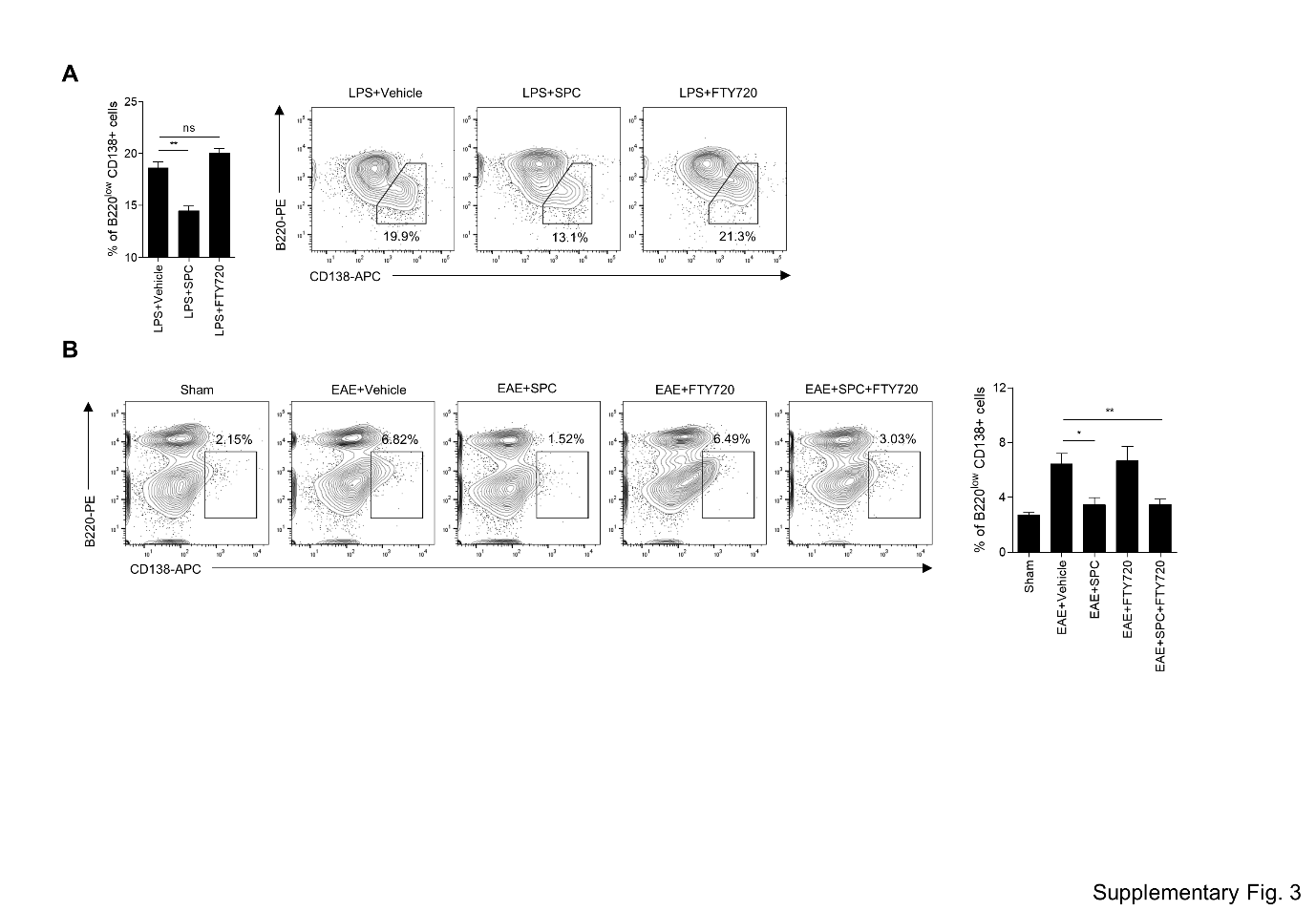
**Supplementary Fig. 4. SPC, but not FTY720, inhibits plasma cell generation.** (A) Mouse B cells were stimulated by LPS (10 μg/ml) with vehicle, SPC (10 μM) or FTY720 (1 μg/ml). B220^low^CD138^+^ plasma cells were compared by flow cytometry. (B) The EAE model was initiated in female C57BL/6 mice. Vehicle, SPC (4 mg/kg) or FTY720 (5 mg/kg) were subcutaneously injected into EAE mice. Plasma cell populations of each group were compared by flow cytometry. Representative dot plots are shown at B, left. *P* values were calculated by Student’s *t*-test. **P* < 0.05, ***P*<0.01, ns, not significant.


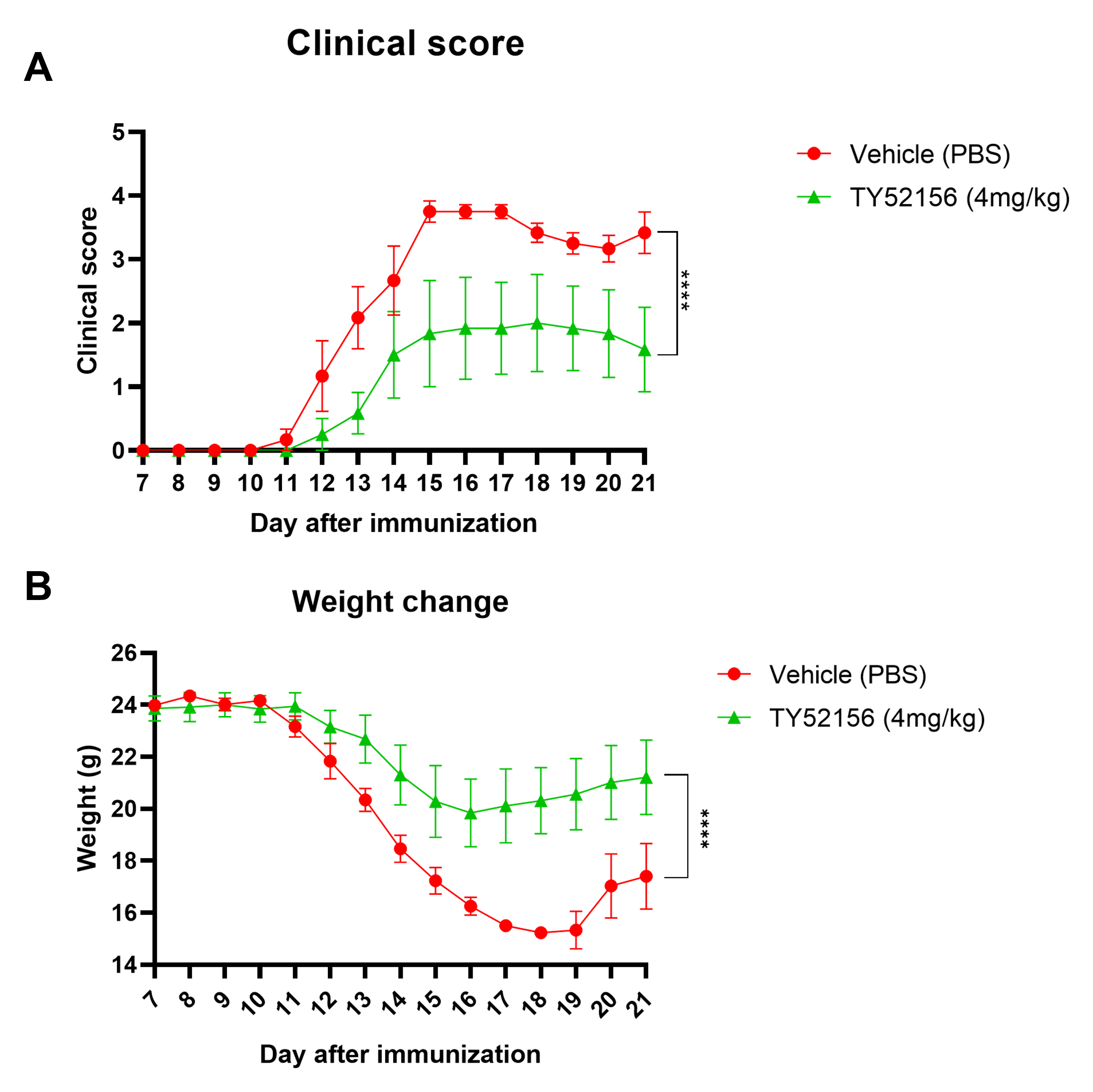


**Supplementary Fig. 5. TY52156 shows therapeutic effects on EAE.**

8-12 week-old female C57BL/6 mice were immunized with MOG^35-55^ / CFA emulsion. Vehicle (PBS) and TY52156 (4 mg/kg) was subcutaneously injected daily. Weight change and clinical score were monitored for 21 days. Data are expressed as the mean ± SEM (n=5-6). *P* values were calculated by two-way ANOVA with Tukey’s multiple comparisons test. *****P* < 0.0001.
